# Discovery of Rift Valley fever virus natural pan-inhibitors by targeting its multiple key proteins through computational approaches

**DOI:** 10.1038/s41598-022-13267-1

**Published:** 2022-06-03

**Authors:** Israr Fatima, Sajjad Ahmad, Mubarak A. Alamri, Muhammad Usman Mirza, Muhammad Tahir ul Qamar, Abdur Rehman, Farah Shahid, Eid A. Alatawi, Faris F. Aba Alkhayl, Wafa Abdullah Al-Megrin, Ahmad Almatroudi

**Affiliations:** 1 Department of Bioinformatics and Biotechnology, Government College University Faisalabad, Faisalabad, Pakistan; 2 Department of Health and Biological Sciences, Abasyn University, Peshawar, Pakistan; 3 Department of Pharmaceutical Chemistry, College of Pharmacy, Prince Sattam Bin Abdulaziz University, Al-Kharj, Saudi Arabia; 4 Department of Chemistry and Biochemistry, University of Windsor, Windsor, Canada; 5 College of Life Science and Technology, Guangxi University, Nanning, China; 6 Department of Medical Laboratory Technology, Faculty of Applied Medical Sciences, University of Tabuk, Tabuk, 71491 Saudi Arabia; 7 Department of Medical Laboratories, College of Applied Medical Sciences, Qassim University, Buraydah, 51452 Saudi Arabia; 8 Department of Pharmaceutical Chemistry and Pharmacognosy, College of Dentistry and Pharmacy, Buraydah Colleges, Buraydah, 51418 Saudi Arabia; 9 Department of Biology, Faculty of Science, Princess Nourah Bint Abdulrahman University, Riyadh, 11671 Saudi Arabia

**Keywords:** Computational biology and bioinformatics, Drug discovery

## Abstract

The Rift Valley fever virus (RVFV) is a zoonotic arbovirus and pathogenic to both humans and animals. Currently, no proven effective RVFV drugs or licensed vaccine are available for human or animal use. Hence, there is an urgent need to develop effective treatment options to control this viral infection. RVFV glycoprotein N (GN), glycoprotein C (GC), and nucleocapsid (N) proteins are attractive antiviral drug targets due to their critical roles in RVFV replication. In present study, an integrated docking-based virtual screening of more than 6000 phytochemicals with known antiviral activities against these conserved RVFV proteins was conducted. The top five hit compounds, calyxin C, calyxin D, calyxin J, gericudranins A, and blepharocalyxin C displayed optimal binding against all three target proteins. Moreover, multiple parameters from the molecular dynamics (MD) simulations and MM/GBSA analysis confirmed the stability of protein–ligand complexes and revealed that these compounds may act as potential pan-inhibitors of RVFV replication. Our computational analyses may contribute toward the development of promising effective drugs against RVFV infection.

## Introduction

The Rift Valley fever is an emerging mosquito and aerosol borne disease that caused by Rift Valley fever virus (RVFV) and associated with endemic to sub-Saharan Africa and Arabian Peninsula^1^. The RVFV (*genus Phlebovirus, family Bunyaviridae*) is an infectious pathogen that can cause disease ranging from a mild illness to hemorrhagic fever and encephalitis in humans^1^. Livestock such as cattle, goats and sheep are also susceptible to RVFV infection^2^. Currently, the options to treat RVFV infected individuals and livestock are limited. The antiviral agent, Ribavirin, was used during past outbreaks, however, its use was limited due to undesirable side effects and the high potential to cause birth defects^3^. Additionally, favipiravir has been also proposed as a broad-spectrum inhibitor of viral hemorrhagic fever^4^. However, the efficacy of drugs in RVFV infected humans or livestock have not yet been verified. The paucity of licensed drugs needed to treat RVFV infection as well as the ability of RNA viruses to mutate and develop resistance to drugs emphasize the continued need for identification of anti-viral agents.

**Figure 1:**
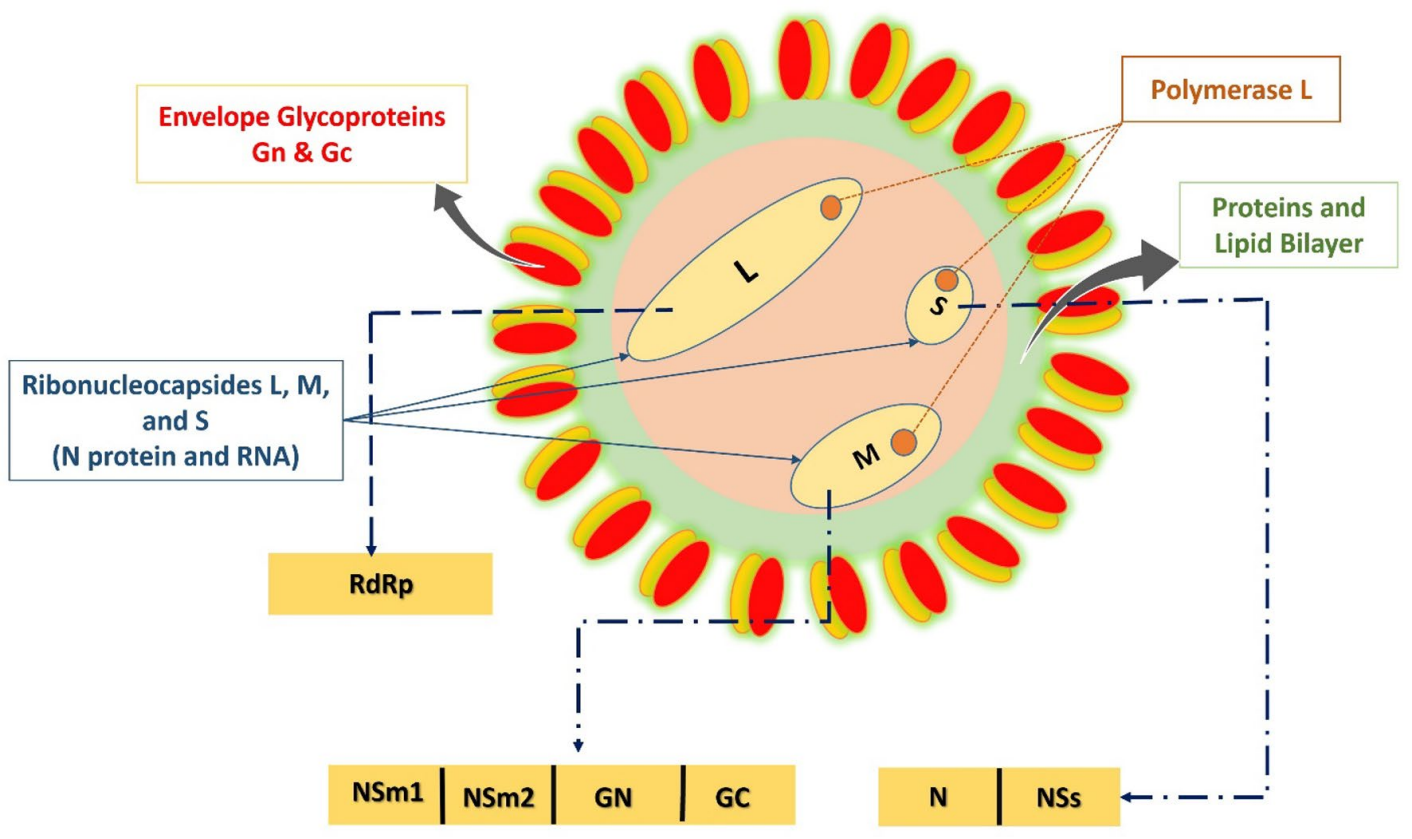
Virion and genome structure of RVFV.

Like other bunyavirus family members, the RVFV genome consists of three negative-sense RNA segments labelled as Large (L), Medium (M) and Small (S) segment^5^. The L segment contains the viral RNA-dependent-RNA polymerase (RdRp) that is essential for viral replication cycle^6^. The M segment encodes the two major structural glycoproteins, referred to as glycoprotein N (GN), glycoprotein C (GC) as well as two nonstructural (NS) proteins, NSm1 and NSm2^7^. The structural glycoproteins, GN and GC, of RVFV, assemble around the outer lipid envelope of RVFV and are required for host cell entry^8,9^. The viral entry into the host cell depends mainly on the ability of these glycoproteins to bind host cellular proteins and to efficiently prompt fusion of the virus envelope with the host cell membrane. They are usually the prime targets of neutralizing antibodies^10^. The S segment, on the other hand, encodes the nonstructural NSs protein, a major RVFV’s virulence factor, and the nucleocapsid (N) protein^11,12^. The RVFV N protein is a 27 kDa protein and encapsulates the RVFV genome by coating the viral RNA^9^ (Fig. 1).

The encapsulation process acts as a shield to protect the viral RNA and prevents the activation of the host anti-viral response by the formation of double-stranded RNA during replication. Therefore, the N protein is essential for several steps in viral replication and transcription cycle^9,12,13^. The N protein is also involved in the virus assembly via the interactions with the glycoproteins (GN and GC)^9^. The blocking of viral fusion activity by targeting viral glycoproteins (GN and GC) as well as the inhibition of viral nucleocapsid N protein function represent an attractive antiviral therapeutic strategy due to their essential role in the viral life cycle.

The use of computational approaches to discover small molecules has become increasingly important in early drug development in recent years^14–18^. Moreover, molecular docking is a widely used tool for prediction of the interaction mechanism between ligands and the target protein^17,19–24^. Putative antiviral compounds have already been found using hierarchical virtual screening approaches against a wide spectrum of viruses including influenza^25^, Ebola^18,26^, Zika^27,28^, human immunodeficiency virus (HIV)^29^, hepatitis C virus HCV^16^ and Dengue Fever^30,31^. MD simulations, which are relied on a general model of the physics governing interatomic interactions, predict how each atom in a protein or other molecular system will move over time^32^. MM/PBSA and MM/GBSA have already been extensively used in biomolecular studies including protein–ligand binding, protein folding, protein–protein interaction, and so on^33^. The prediction of ADMET properties is critical in the drug design process because these properties are responsible for the failure of approximately 60% of all drugs in the clinical phases^34^ This research revolves around the sequential computational screening methods including structure-based virtual screening, MD simulations, MMGBSA calculation and ADME-T analysis of ~ 6000 phytochemicals against three key RVFV structural proteins (GN, GC, and N).

## Material and methods

### Proteins preparation

The x-ray structures of Glycoprotein (C) (PDB ID: 4HJC), Glycoprotein (N) (PDB ID: 6F8P) and Nucleocapsid (N) (PDB ID: 3OV9) were retrieved from RCSB Protein Data Bank^35^. The X-ray diffraction analysis demonstrated that all these three structures have resolution up to 4.15 Å, and 1.60 Å, and 1.60 Å, respectively. The Molecular Operating Environment (MOE) was used to prepare the protein structures for docking. Mainly, refinement of structures such as removal of H_2_O molecules, 3D protonation and energy minimization were performed through MOE using default parameters^36^. Minimized structures were further used for molecular docking.

### Ligand database preparation

A library of ~ 6000 phytochemicals was prepared by collecting phytochemicals with known antiviral activity from several databases such as PubChem, MAPS, MPD3 and ZINC in .sdf format^37–39^. Energy minimization for each ligand was performed using the following parameters, Gradient: 0.05, Force Field: MMFF94X and Chiral Constraint: Current Geometry. The minimized ligands were then saved into the MOE database in .mol format.

### Molecular docking

The phytochemicals were docked against GC, GN, and N proteins using MOE Dock tool while setting the specific docking sites^36^. Site Finder tool was used to predict the active sites of GC, GN, and N proteins^40–42^. MOE possesses multiple docking algorithms to get the best poses of docked complexes. In this analysis, triangular matcher algorithm was utilized with ten iterations to get the best poses for further analysis^43^. Docking binding scores were utilized as a key evaluation criterion to filter out promising compounds. For each docked complexes, the model with the maximum absolute value of binding energy were considered accurate.

### Receptor ligand interaction analysis

Two dimensional (2D) diagram of protein–ligand complexes were obtained using LigX tool in MOE to clearly visualize the ligand-receptor interaction of best-docked complexes^44^. LigX tool in MOE creates a 2D graph showing interacting forces (such as covalent and non-covalent interaction) for compounds within the active sites of RVFV proteins. Later, 3D diagram of best-docked complexes were displayed using PyMOL and Discovery Studio^36,45^.

### Drug scan/ADME toxicity

The analysis of the drug-likeness as well as ADME-T (absorption, distribution, metabolism, and excretion—toxicity) properties of drug molecules is a crucial phase in the drug discovery pipeline^46^. Theses parameters were determined using the canonical simplified molecular input line-entry system (SMILES) of each molecule as input file. The drug likeliness of docked molecules was calculated using the drug scan tools at Molinspiration web-server following "Lipinski's Rule of Five" criteria^47,48^. Furthermore, the ADME properties were predicted using ADMETlab 2.0 and Swiss ADME webservers. The Protox II webserver was used to predict the acute oral toxicity of molecules^49^. This server categori*z*ed compounds into six toxicity classes (1–6), with class 1 being the most dangerous and poisonous, with an estimated fatal dose (LD50) of less than 5, and class 6 denoting non-toxicity with an LD50 > 5000.

### Molecular dynamics simulation

Molecular Dynamic (MD) simulation is a critical computational approach for investigating the structural stability and dynamics of docked complexes. The AMBER18^50^ software was used to perform MD simulations of the antiviral compounds in complex with target proteins. The top docked complexes were deliberately solvated with H_2_O molecules, and then counter ions were added in order to create a neutral system. The TIP3P solvent model was then used to generate a water box with a thickness of 12 Å to encircle the complexes^51^. The docked complexes were simulated by employing periodic boundary conditions^32^. Further, for non-bounded interactions, a boundary value of 8 Å was set. After minimizing water molecules for 500 cycles, the entire system was minimized for 1000 rounds. The temperature of each system was then steadily increased to 300 K. The systems were equilibrated for 100 ps using the NPT ensemble. During the equilibration of counter ions and water molecules, solutes in the first phase were restricted for 50 ps, and protein side chains were then relaxed. A 100 ns MD simulation was run for two fs at 300 K and 1 atm using the NPT ensemble. The SHAKE algorithm^52^ was employed to restrain the hydrogen and covalent bonding, while Langevin dynamics^53^ were used to regulate system temperature. The initial structure was employed as a baseline, and AMBER's CPPTRAJ^54,55^ was used to generate a RMSD plot to ensure that the system MD simulation was converging^56^. The structural flexibilities of ligands were determined using the ligand RMSD method^57^. RoG was studied for the compactness and three-dimensional packaging of the complex. The RMSF reflects the average root mean square distance between an atom and its average geometric position in a certain dynamics^58^.

### Binding free energy calculations

The MM-GBSA method, implemented in AMBER 18, was used to calculate the binding free energies (ΔG_tol_) of RVFV proteins complexed with the most potential hit compounds^59,60^. Briefly, 10,000 snapshots were generated from the last 20 ns stable trajectories with a 2 ps interval for each system. The total binding free energy is calculated as the solvation free energy (ΔG_sol_) and the sum of the molecular mechanics binding energy (ΔE_MM_), as shown below.

$$\begin{gathered} \Delta {\text{E}}_{{{\text{gas}}}} = \, \Delta {\text{E}}_{{{\text{ele}}}} + \Delta {\text{E}}_{{{\text{int}}}} + \Delta {\text{E}}_{{{\text{vdw}}}} \hfill \\ \Delta {\text{G}}_{{{\text{sol}}}} = \, \Delta {\text{GN}}_{{\text{p}}} + \Delta {\text{G}}_{{\text{p}}} \hfill \\ \Delta {\text{G}}_{{{\text{tol}}}} = \, \Delta {\text{G}}_{{{\text{sol}}}} + \Delta {\text{E}}_{{{\text{MM}}}} \hfill \\ \end{gathered}$$

where, ΔE_MM_ is further divided into electrostatic energy (ΔE_ele_), internal energy (ΔE_int_) and van der Waals energy (ΔE_vdw_). The sum of non-polar (ΔGN_p_) and polar (ΔG_p_) components contribute to the total solvation free energy (Gsol). The MM-GBSA method is well demonstrated in binding free estimation for antiviral inhibitors^29,61–63^.

## Results and discussion

### Molecular docking

**Figure 2:**
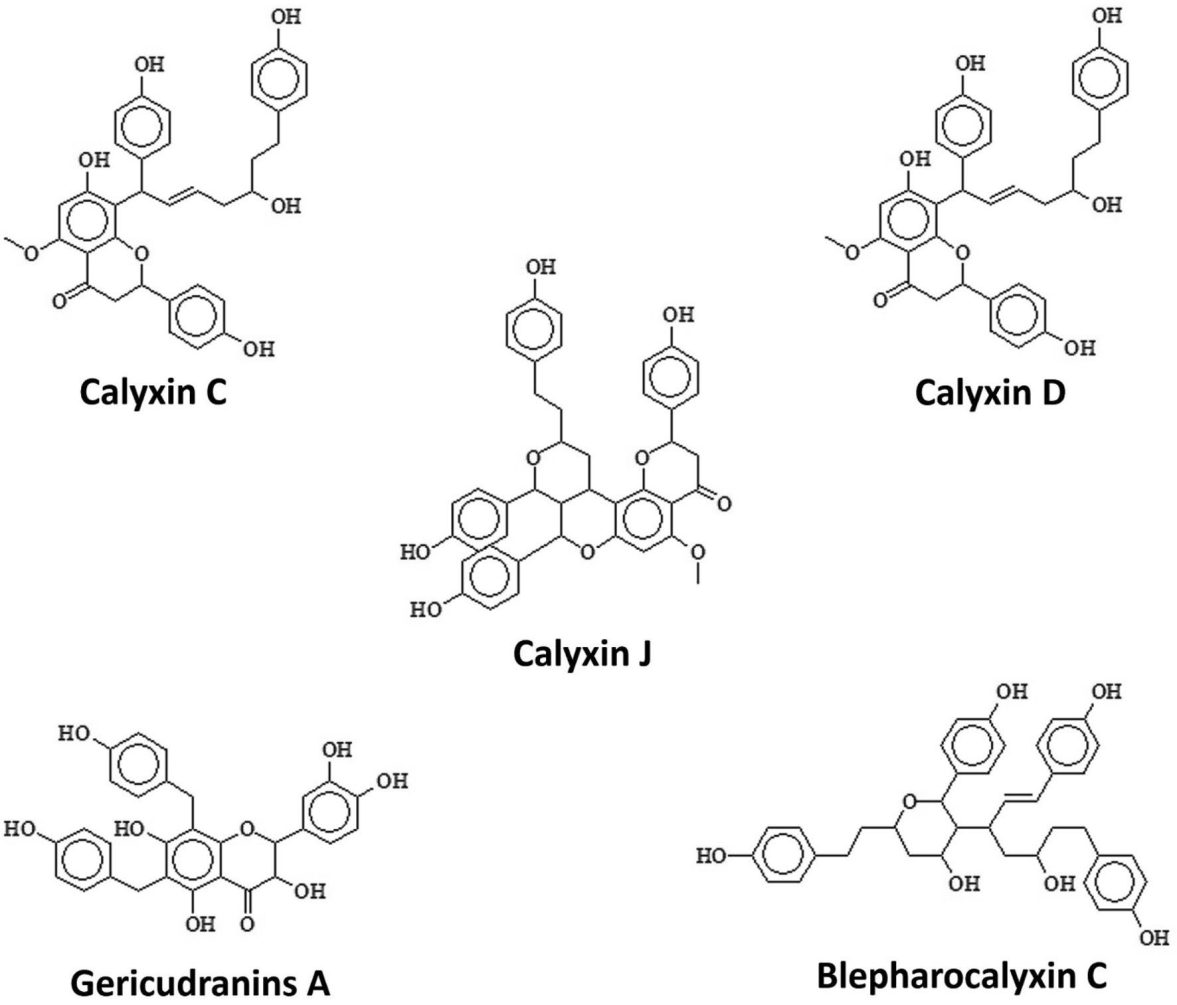
Two-dimensional presentation of top compounds binding to RVFV proteins.

The developed phytochemicals database was docked against RVFV proteins (GN, GC, and N). Molecular docking is a technique for predicting how ligands will bind to their protein targets. As a result, molecular docking become an important tool in virtual screening and the development of novel antiviral medicines to combat severe disorders^30,64^. Docked compounds were selected by applying a strict filtering criterion that took into account following conditions: strength of H-bond interaction, binding pocket maximum occupancy with the lowest Gibbs free energy and docking score/strength compared to reported native ligand. Reported native ligands 1,2-ethanediol; 3-Aminophthalylhydrazido-*N*-acetyl-beta-glucosaminide; and Nitrite Ion of RVFV GN, GC and N proteins, respectively, were used as control and only those compounds which showed stronger binding affinity were chosen for further analyses. Calyxin C, calyxin D, calyxin J, gericudranins A, and blepharocalyxin C (Fig. 2) were discovered, binding with the interacting residues of all 3 target proteins at high binding affinity (Table 1).

**Table 1:** Properties profile of candidate compounds and controls*.*

PubChem ID	Phytochemicals name	Glycoprotein N				Glycoprotein C				Nucleocapsid			
		Docking score	Binding Affinity (kcal/mol)	Inhibition constant (Ki)	Interacting residue	Docking score	Binding Affinity (kcal/mol)	Inhibition constant (Ki)	Interacting residue	Docking score	Inhibition constant (Ki)	Binding affinity (kcal/mol)	Interacting residue
10460896	Calyxin C	−12.32	−11.44	78.10 µM	ARG-461, LYS-199, Leu-299, Lys-247	−14.95	−12 to 12	51.24 µM	Arg-810, Arg-949	−18.02	32.88 µM	−14.27	Arg-B70, Arg-B185, Gln-A198
10008443	Calyxin D	−11.30	−11.26	71.01 µM	HIS-249, ASP-301, Lys-247, ARG-461	−10.68	−12.39	68.67 µM	Arg-810, Lys-813	−16.51	35.98 µM	−13.69	Arg-B185, Lys-B67, Arg-B70
42608060	Calyxin J	−14.15	−11.44	55.17 µM	ARG-461, LYS-199, Lys-247	−11.45	−13.12	58.112 µM	Arg 949, Glu 811, Leu 789	−17.72	28.90 µM	−14.24	Arg-B64, Lys-B74, Arg-B70
10436583	Gericudranins A	−11.74	−12.85	68.67 µM	LYS-199, HIS-249, Leu-199, Leu-299	−13.39	−12.85	−48.62 µM	Arg-949, Lys-813, Asn-592, Glu-811, Asp-793	−17.13	45.33 µM	−14.99	Arg-B70, Arg-B64
101065840	Blepharocalyxin C	−10.96	−11.65	95.67 µM	LYS-199, GLU-196, Lys-247, HIS-249, Leu-299	−12.10	−10.69	−52.64 µM	Lys-813, Asp-793	−16.72	41.87 µM	−14.57	Arg-B70, Lys-B67, Arg-B64
**Native/reference ligands**													
174	1,2-ethanediol	−6.22	−5.70	150.71 µM	His-249 Leu-299	–	–	–	–	–	–	–	–
195591	3-Aminophthalylhydrazido-*N*-acetyl-beta-glucosaminide	–	–	–	–	−9.05	−11.42	55.007 µM	Thr-796 His-836 Arg-810	–	–	–	–
946	Nitrite ion	–	–	–	–	–	–	–	–	−6.85	101 µM	−5.12	Arg-B64

**Figure 3:**
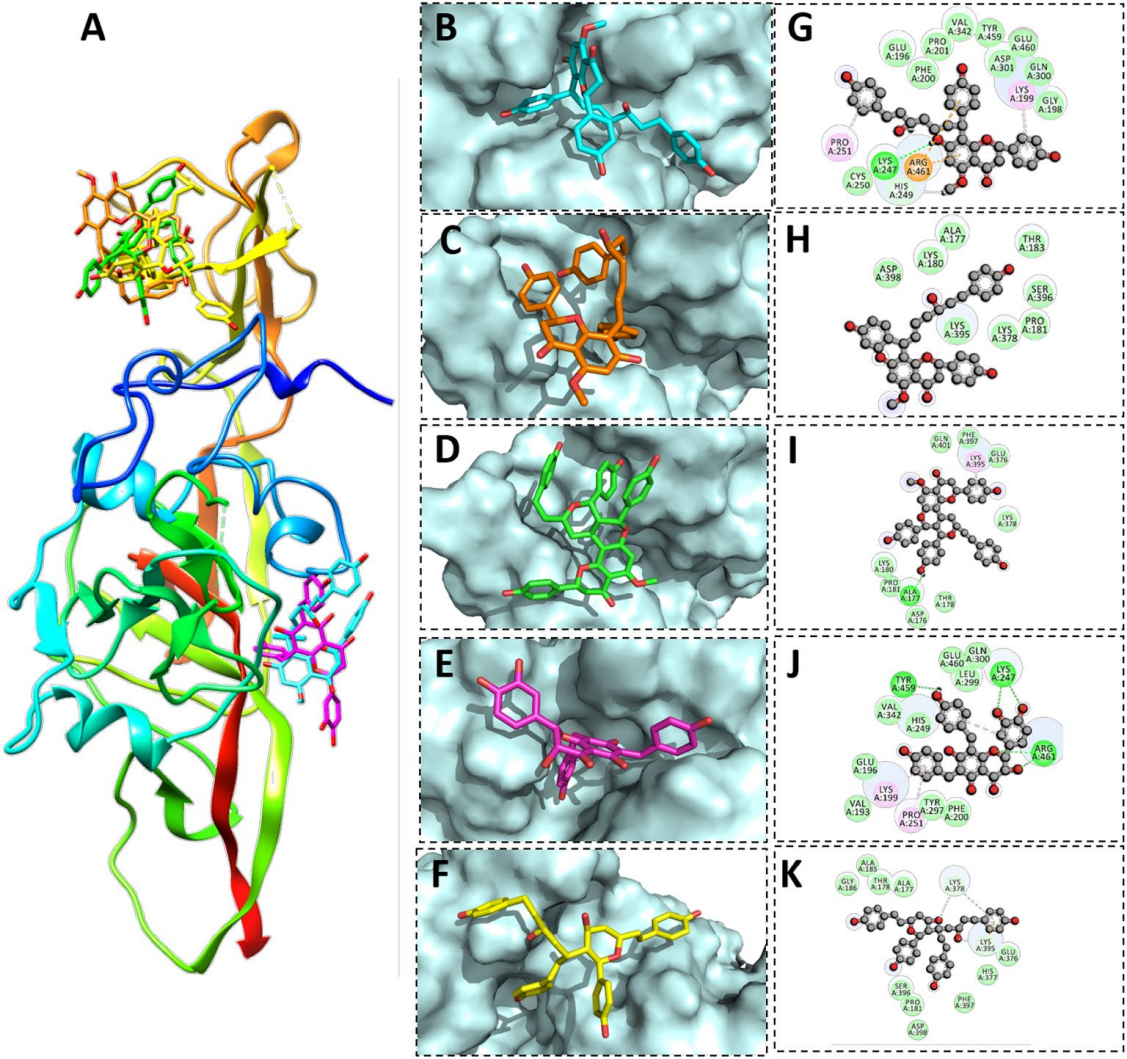
Binding modes and interaction mechanisms of novel GN protein inhibitors. (**A**) Inhibitory binding modes of all ligands. A 3D close view into the binding mode of calyxin C (**B**), calyxin D (**C**), Calyxin J (**D**), Gericudranins A (**E**) and Blepharocalyxin C (**F**). 2D interaction analysis of calyxin C (**G**), Calyxin D (**H**), Calyxin J (**I**), Gericudranins A (**J**) and Blepharocalyxin C (**K**).

Calyxin C was bound to GN protein with a score of -12.32 kJ/mol, forming hydrogen bonds with the side chains of Arg-461, Lys-199, Leu-299 and Lys-247 and calyxin D was bound with a binding score of −11.30 kcal/mol, forming hydrogen bonds with side chains of His-249, Asp-301, Lys-247 and Arg-461. Calyxin C and calyxin D showed strong binding with GN active residues followed by calyxin J, gericudranins A, and blepharocalyxin C with binding scores of −14.15 kcal/mol, −11.74 kcal/mol, and −10.96 kcal/mol, respectively (Table 1). Except for gericudranins A, all ligands showed strong hydrogen bonding with the conserved Lys-247 (Fig. 3).

**Figure 4:**
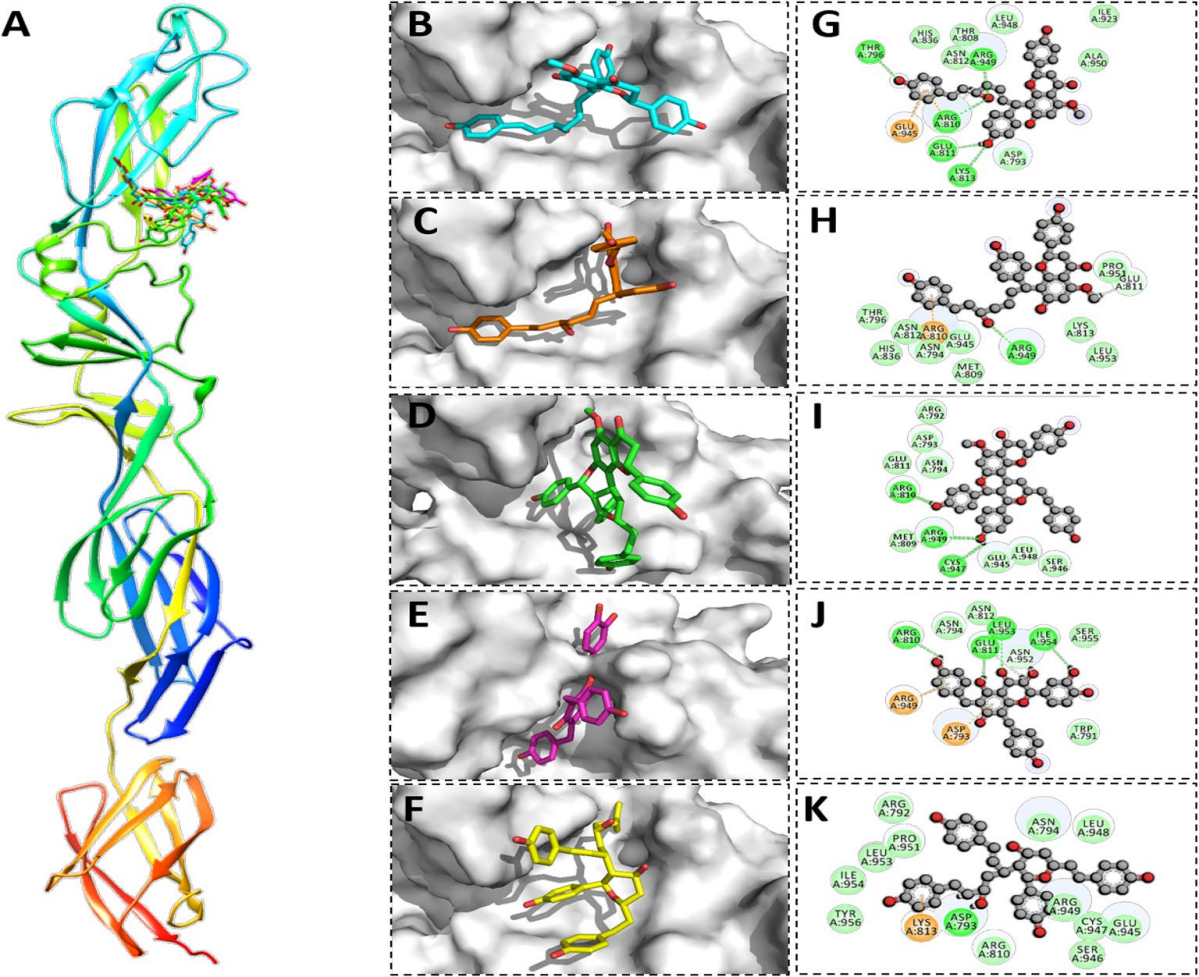
Binding modes and interaction mechanisms of novel GC protein inhibitors. (**A**) Inhibitory binding modes of all ligands. A 3D close view into the binding mode of calyxin C (**B**), calyxin D (**C**), calyxin J (**D**), Gericudranins A (**E**) and Blepharocalyxin C (**F**). 2D interaction analysis of calyxin C (**G**), Calyxin D (**H**), Calyxin J (**I**), Gericudranins A (**J**) and blepharocalyxin C (**K**).

Likewise, in GC protein calyxin C, calyxin D, calyxin J, gericudranins A, and blepharocalyxin C have been found to bind through significant hydrogen bonds having binding scores of −14.95 kcal/mol, −10.68 kcal/mol, −11.45 kcal/mol, −13.39 kcal/mol, and −12.10 kcal/mol respectively. All the essential residues (Arg-810, Arg-949, and Lys-813) that comprise the active site was found to serve as electron donors in the formation of a H-bond network. Other active site residues (Leu-789, Asn-592, Asp-793) showed strong non-covalent and hydrophobic interactions, as detailed in Table 1 and illustrated in Fig. 4.

**Figure 5:**
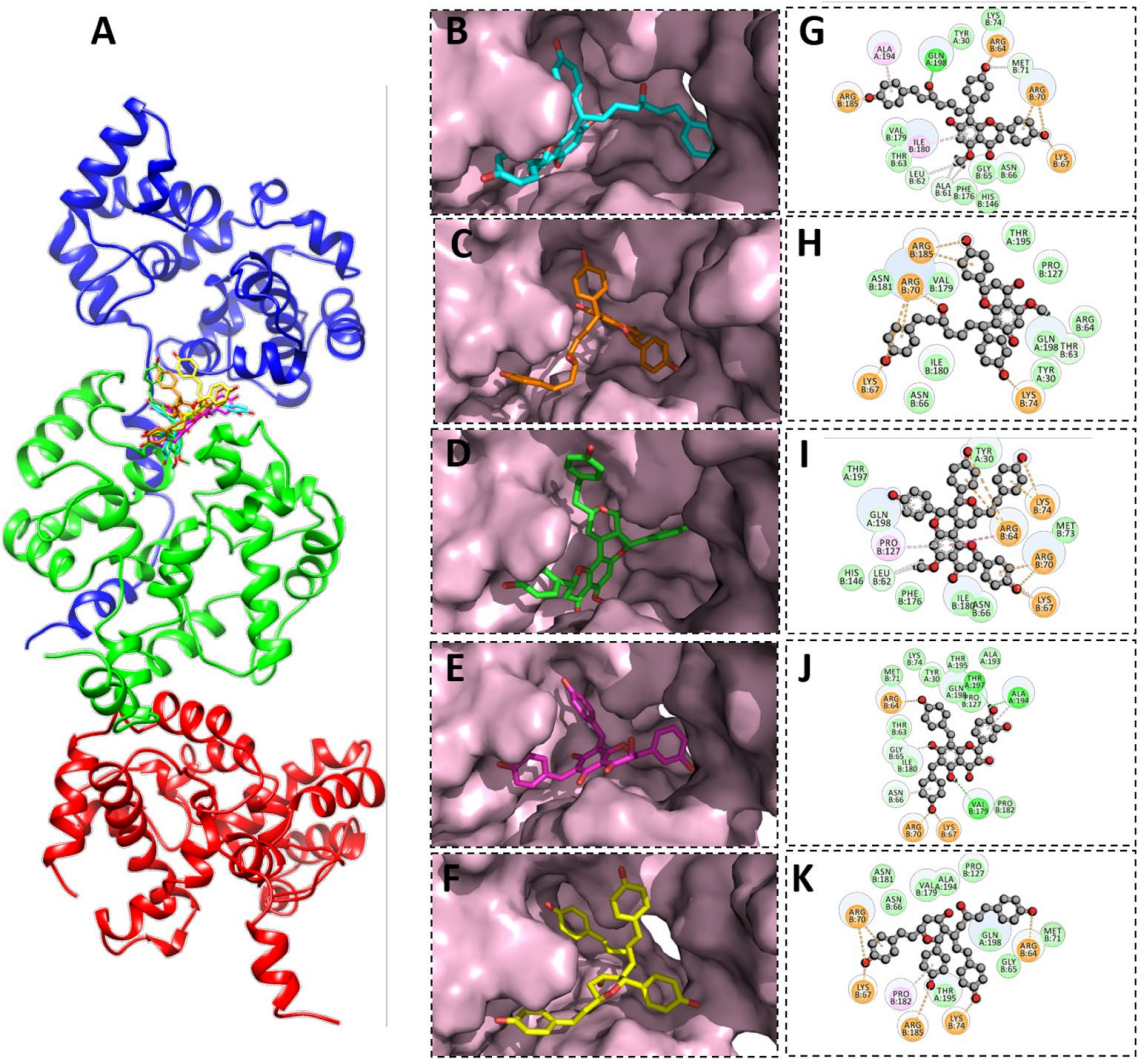
Binding modes and interaction mechanisms of novel N protein inhibitors. (**A**) Inhibitory binding modes of all ligands. A 3D close view into the binding mode of calyxin C (**B**), Calyxin D (**C**), Calyxin J (**D**), Gericudranins A (**E**) and Blepharocalyxin C (**F**). 2D Interaction analysis of calyxin C (**G**), Calyxin D (**H**), Calyxin J (**I**), Gericudranins A (**J**) and blepharocalyxin C (**K**).

Similarly, top five inhibitors (calyxin C, calyxin D, calyxin J, gericudranins A, and blepharocalyxin C) which were found to inhibit glycoproteins (GN and GC) were also observed as inhibiting N protein. The binding energies of the five active compounds were in the range of −18.02 kcal/mol to −16.72 kcal/mol (Table 1). Most compounds established hydrogen bonds with Arg B64, Lys B74, and Arg B70, which indicates that these compounds have potential role to play in disease management. Hydrogen interactions between the side chains and backbone atoms of these N protein residues stabilized the inhibitors spatially within the pocket. All ligands showed strong hydrogen bonding with the conserved Arg B70 (Fig. 5).

All top five inhibitors were making strong bonds with functionally and structurally key interacting sites of the RVFV proteins. However, calyxin C was ranked first because it had the highest binding score and affinity. The compounds discovered in present study may have synergistic or additive effects against RVFV. This is an important aspect in case of viruses, which are constantly evolving due to a higher mutation rate. In case of HCV and HIV infections, the advantages of synergistic treatment techniques have already been documented^65,66^.

### Drug scan/ADME toxicity

The drug likeness (DL) properties of final active compounds were identified using Molinspiration server which offers ADMET tool to scan the pharmacokinetic properties. The rule states that a compound must contain less than five H-bond donors, less than ten H-bond acceptors, a molecular mass of less than five hundred daltons, and log P should be greater than five. Drug-likeness properties of potential compounds are enlisted in Table 2. According to the results, all compounds showed only one violation of Lipinski rule of five i.e., MW > 500 daltons.

**Table 2:** Lipinski’s rule of five DL properties of potential compounds.

Compounds	MW	HBA	HBD	A log *P*
Calyxin C	578.62	8	1	3.15
Calyxin J	683.73	9	1	4.34
Gericudranins A	514.49	9	5	0.86
Blepharocalyxin C	606.72	7	2	4.21
Calyxin D	578.62	8	1	3.15

Additional analyses were performed on the ADMET properties of selected compounds. The assessment of compounds' ADMET properties is a key step in the drug discovery toolbox. A major portion of proposed drug candidates failed to reach the final step because of toxicity and poor pharmacokinetic properties^66,67^. ADMET lab 2.0 and Swiss ADMET were used to predict ADMET properties of screened compounds, and their results are presented in Table 3.

**Table 3:** ADMET profiling of best docked compounds.

Parameters	Compounds				
	Calyxin C	Calyxin D	Calyxin J	Gericudranins A	Blepharocalyxin C
**Absorption**					
BBB	No	No	No	No	No
GI absorption	Low	Low	low	Low	Low
Caco-2 permeability	−6.266	−5.356	−5.727	6.57	6.269
Human oral bioavailability	0.55	0.55	0.55	0.17	0.17
Log P	3.15	3.15	4.34	0.86	4.21
TPSA (Å^2^)	136.68	136.68	134.91	167.91	130.61
**Metabolism**					
P-glycoprotein substrate	Yes	No	No	Yes	Yes
P-glycoprotein inhibitor	No	Yes	No	No	Yes
CYP450 2C9 substrate	Yes	Yes	No	No	No
CYP450 2D6 substrate	Yes	Yes	Yes	No	No
CYP450 3A4 substrate	Yes	Yes	No	No	Yes
CYP450 1A2 inhibitor	No	No	No	No	No
CYP450 2C9 inhibitor	Yes	No	Yes	Yes	Yes
CYP450 2D6 inhibitor	No	No	No	No	No
CYP450 2C19 inhibitor	No	No	No	No	No
CYP450 3A4 inhibitor	Yes	No	Yes	Yes	Yes
**Toxicity**					
AMES Toxicity	Non-toxic	Non-toxic	Non-toxic	Non-toxic	Non-toxic
Carcinogens	Non-carcinogenic	Non-carcinogenic	Non-carcinogenic	Non-carcinogenic	Non-carcinogenic
Acute oral toxicity	165.732 mg/kg	(165.732 mg/kg)	368.81 mg/kg	217.306 mg/kg	285.011 mg/kg

Drug distribution and absorption of drug molecules are indicated by gastrointestinal absorption (GI) and blood–brain barrier (BBB) permeation^68–70^. Table 3 shows that all compounds have low gastro-intestinal absorption and no BBB permeation. The compounds' absorption was further demonstrated by caco-2 permeability values ranging from −5.35 to −6.57 log unit. In ADMETlab 2.0 server, permeability greater than −5.15 log unit indicates optimal caco-2 absorption. Furthermore, several cytochromes (CYPs) regulate drug metabolism, with CYP2C19, CYP2C9, CYP3A4, CYP1A2, and CYP2D6 being essential for drug molecules biotransformation^71,72^. Furthermore, *p*-glycoprotein inhibitors decrease the bioavailability of drugs known to be transported^73–75^. Calyxin D, and blepharocalyxin C are inhibitors of *p*-glycoprotein while others are non-inhibitors. Similarly, calyxin D and calyxin J are negative substrates of *p*-glycoprotein, while others are substrates, which explain their good absorption profiles. Following that, toxicity prediction research was carried out to assess the compounds' safety profile. All selected compounds were found to be non-toxic and non-carcinogenic. These findings suggested that no toxicophore associated with these compounds and could be developed into safer drugs.

### Molecular dynamics simulation

**Figure 6:**
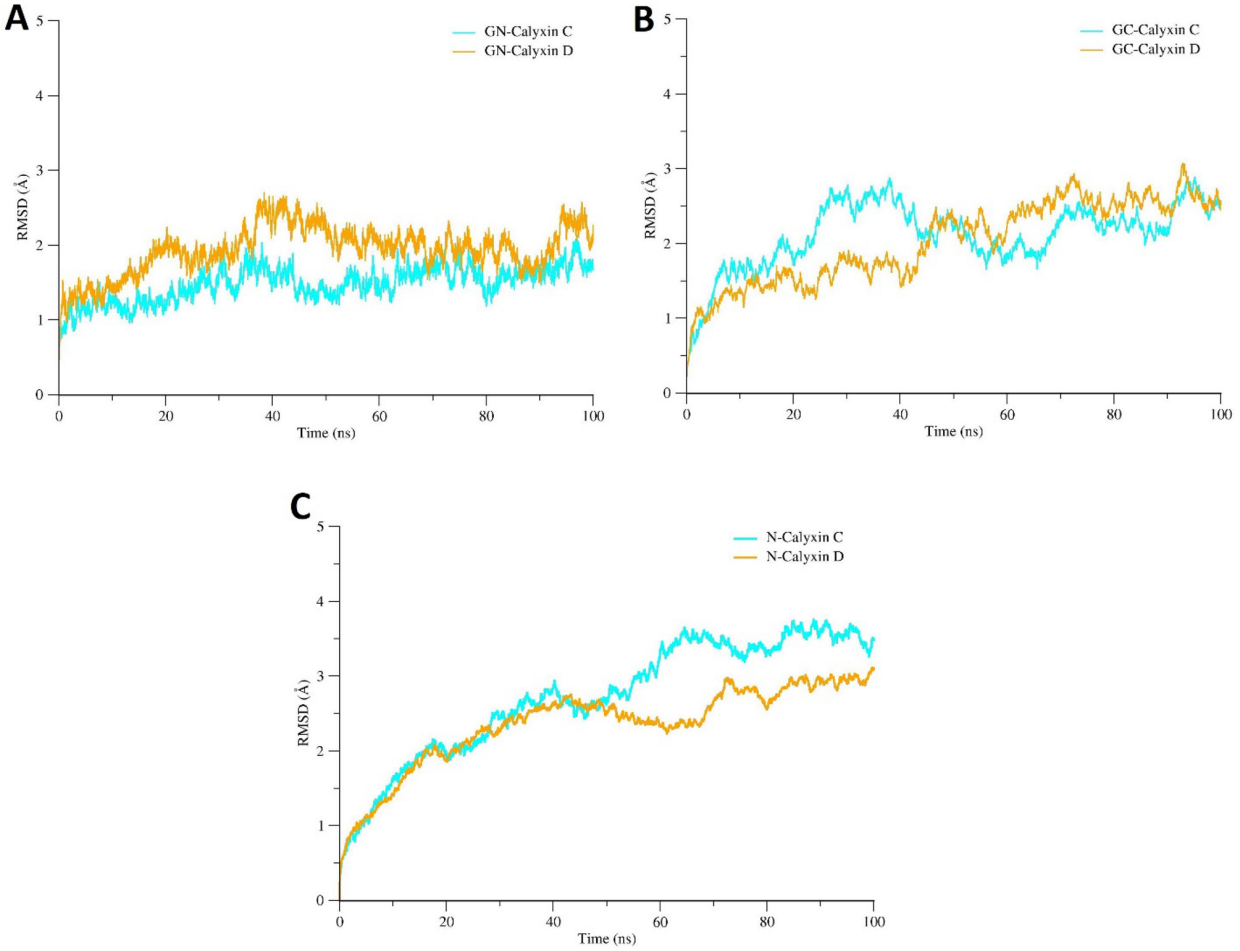
(**A**) Root mean square deviation (RMSD) analysis of RVFV GN in complex with two candidate compounds (calyxin C and calyxin D*)* during 100 ns MD simulation. (**B**) RMSD analysis of RVFV GC in complex with calyxin C and calyxin D. **(C)** RMSD analysis of RVFV N in complex with calyxin C and calyxin D.

MD simulation is a powerful approach in biophysical research that offers important dynamic values of protein–ligand interactions^53,76,77^. A number of studies showed that some systems require MD simulations to discover the accurate binding conformations^21,29,77–81^ and therefore, it has profound importance in computer-aided drug discovery^18,82^. For the present study, MD simulation were carried out on the top models obtained through docking with calyxin C and calyxin D inhibitors. To explicate the dynamic stability and ensure the rationality of the ligand sampling, the RMSD values of GN, GC and N protein, and heavy atoms of calyxin C and calyxin D inhibitors relative to the respective initial structures were calculated, and RMSD trajectories were analyzed over a period of throughout 100 ns. The RMSD plots of protein–ligand complexes are displayed in Fig. 6. RMSD values of complexes were predicted as: GN-calyxin C (maximum, 1.8 Å; mean, 1.2 Å), GN-calyxin D (maximum, 2.5 Å; mean, 1.9 Å), GC-calyxin C (maximum, 2.6 Å; mean, 1.8 Å) , GC-calyxin D (maximum, 2.8 Å; mean, 2.25 Å) , N-calyxin C (maximum, 3.5 Å; mean 2.45 Å) and N-calyxin D (maximum, 2.6 Å; mean, 2.15 Å) (Fig. 6). In terms of 3D structure, all the receptors are relatively stable, and no secondary structure flexibility was observed. Hence, calyxin C and calyxin D binding poses remained unchanged, indicating stable and strong complexes formation. Although the conformations of the complex were expanded, the RMSD remained converge under 3 Å. The RMSD of protein backbone atoms of the order of 1–3 Å with no high conformational change certainly favours that the system is well equilibrated and calyxin C and calyxin D inhibitors binds more stably with the binding pocket of GN, GC and N proteins, which is an acceptable measure in protein–ligand simulation systems^76,83^.

**Figure 7:**
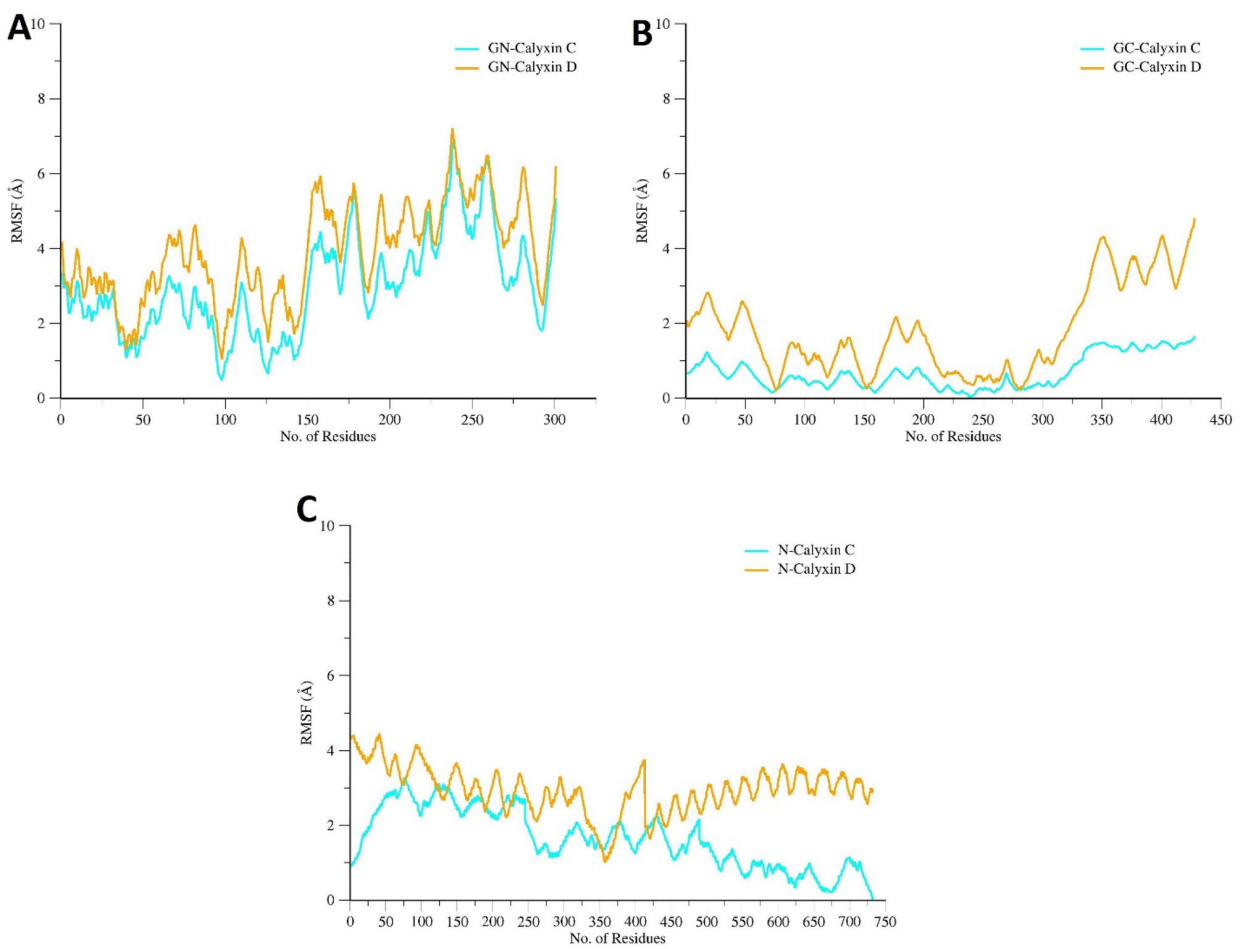
(**A**) Root mean square fluctuation (RMSF) analysis of RVFV GN in complex with two candidate compounds (calyxin C and calyxin D*)* during 100 ns MD simulation. (**B**) RMSF analysis of RVFV GC in complex with calyxin C and calyxin D. **(C)** RMSF analysis of RVFV N in complex with calyxin C and calyxin D.

The stability and residual flexibility of proteins in presence of calyxin C and calyxin D was further computed through RMSF analysis. Mean RMSF for GN- calyxin C is 3.4 Å GN-calyxin D is 3.8 Å, GC-calyxin C is 0.6 Å, GC-calyxin D is 1.7 Å, N-calyxin C is 1.2 Å and N-calyxin D is 2.2 Å (Fig. 7). These values indicate a high level of agreement on intermolecular stability. Generally, the GN-calyxin D shows high rates of fluctuation starting from residue 150 to 250 exhibiting a high tendency to fluctuate.

**Figure 8:**
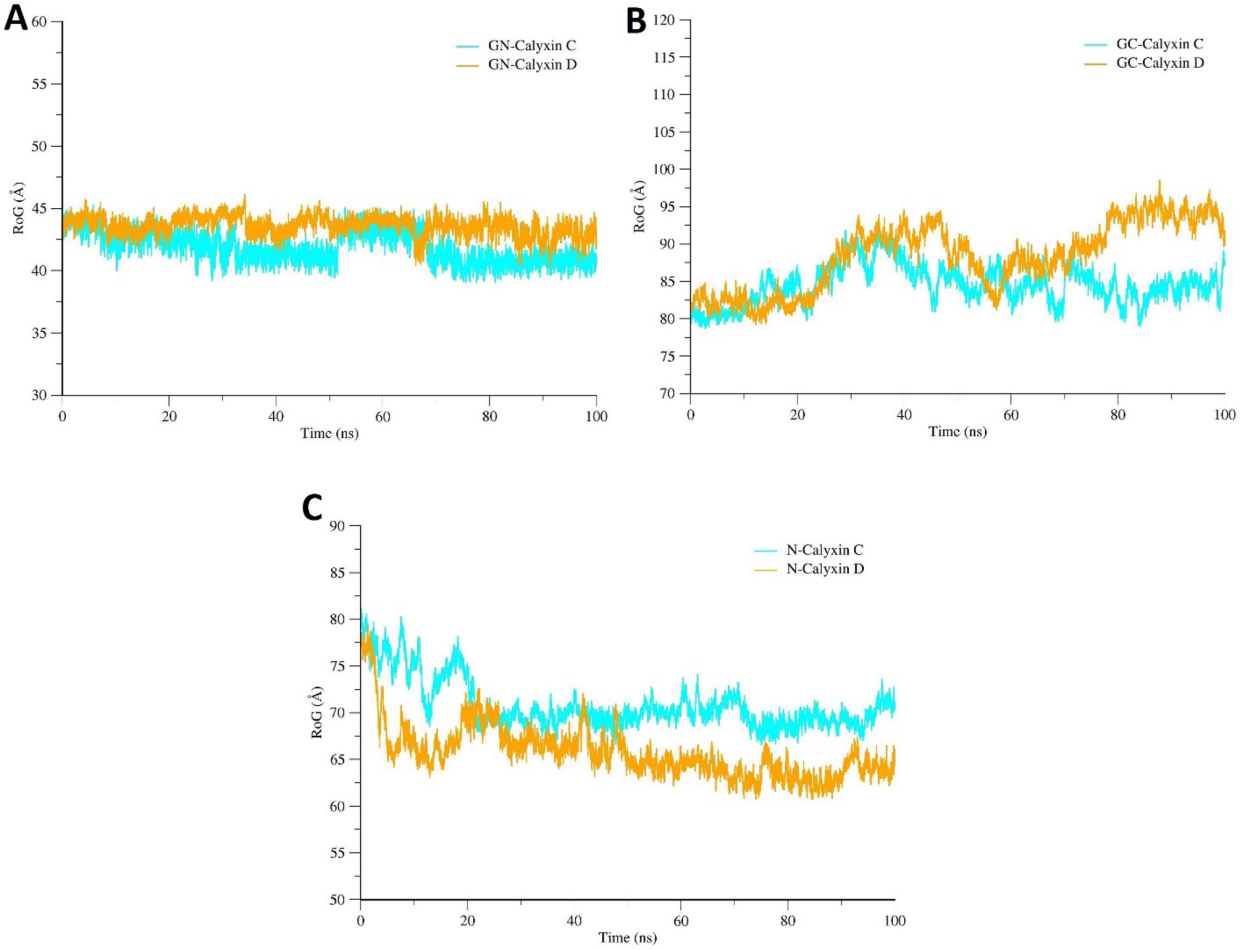
(**A**) Radius of gyration (Rg) analysis of RVFV GN in complex with two candidate compounds (calyxin C and calyxin D*)* during 100 ns MD simulation. (**B**) Rg analysis of RVFV GC in complex with calyxin C and calyxin D. **(C)** Rg analysis of RVFV N in complex with calyxin C and calyxin D.

Furthermore, Rg analysis was conducted to assess structural equilibrium and protein compactness over the simulation time. An optimum Rg value should be low in case of globular proteins, however, the Rg value for protein form with a greater number of turns and loops could be significantly larger^83^. The Rg values of the complexes are follows; GN-calyxin C (maximum, 44.8 Å; mean, 42.5 Å), GN-calyxin D (maximum, 45 Å; mean, 43 Å), GC-calyxin C (maximum, 90 Å; mean, 84 Å), GC-calyxin D (maximum, 94.5 Å; mean, 86 Å), N-calyxin C (maximum, 80 Å; mean, 70 Å) and N-calyxin D (maximum, 77 Å; mean, 67 Å) (Fig. 8). For RVFV GN-calyxin C and GN-calyxin D, both complexes showed compactness, while RVFV GC/N-calyxin C and GC/N-calyxin D complexes indicated slight loss of compactness at the end. Overall, no significant loss in compactness was observed during the simulation period in all complexes.

### Binding free energy calculations

Binding free energies of best-docked complexes with calyxin C and calyxin D inhibitors were calculated by employing MMGBSA methods to better understand the complexes binding ability with RVFV proteins. The binding energies of the complexes are given in detail in the Table 4. The results from MM/GBSA analysis demonstrated the favorable affinity of calyxin C and calyxin D inhibitors within the binding pocket RVFV GN/GC and N proteins. From the calculated results, RVFV GN/GC and N proteins showed more favorable total binding free energy (ΔG_tol_) in complex with calyxin C (−27, −18.64, and −33.54 kcal/mol) as compared to calyxin D (−21.58, −17.92, and −23.54 kcal/mol), respectively. Whereas RVFV GN/GC and N proteins with bound inhibitors showed a considerable increase in negative values for vdW interaction energies (ΔG_vdW_ range from −27.11 to −36.99 kcal/mol) as compared to electrostatic interactions (ΔG_elec_ range from 3.08 to − 45.35 kcal/mol). In protein/ligand systems, vdW interactions (ΔG_vdW_) are considered important in overall strength of nonpolar interactions^84,85^. Among all complexes, vdW highly contributed towards stabilising the complexes with comparatively higher negative values than electrostatic interactions (ΔG_elec_). Besides, polar solvation energy (ΔG_sol_) displayed the energy associated with dissolving calyxin C and calyxin D inhibitors within the solvent, and highly positive polar solvation energies (ΔG_sol_ ranges from 13 to 60.17 kcal/mol) were obtained, which was demonstrated to be unfavorable. The more favorable total binding free energy in complex with calyxin C determined a more stable protein–ligand interaction profile within the binding site GN/GC and N proteins, which was evident from the less residual flexibility compared to calyxin D (Fig. 7).

**Table 4:** Binding free energy components of RVFV proteins.

Energy component	Glycoprotein N		Glycoprotein C		Nucleocapsid	
	Calyxin C	Calyxin D	Calyxin C	Calyxin D	Calyxin C	Calyxin D
Van der Waals	−36.78	−36.66	−36.99	−27.11	−44.71	−36.03
Electrostatic	−45.35	−16.72	−27.89	−29.88	3.74	3.08
Polar solvation	60.17	36.90	52.45	43.22	13.00	13.23
Non-polar solvation	−5.04	−5.09	−6.20	−4.14	−5.57	−4.09
Net gas phase	−82.13	−53.39	−64.88	−56.99	−40.97	−32.95
Net solvation	55.12	31.81	46.24	39.07	7.43	9.41
Net complex energy	−27.00	−21.58	−18.64	−17.92	−33.54	−23.54

## Conclusions

RVFV is a pathogenic agent and associated with hemorrhagic fever and liver damage. Previous studies reported that few antiviral drugs such as; benzavir-2, favipiravir T-705, and 2′-fluoro-2′-deoxycytidine (2′-FdC), have shown anti-RVFV activities and currently are under development process^86–89^. However, no proven RVFV drug or licensed vaccine are available to date in market. Natural molecules-based drug discovery through a pipeline of modern computational tools could be an essential framework towards identifying potential hits against RVFV infection. As an initial step, we designed this study and utilized an integrated computational approach, that identified five novel hit compounds from a focused library of 6000 natural compounds, bearing specific scaffolds which can inhibit the crucial proteins (GN, GC and N) of RVFV. Our discovered drug-like molecules, including calyxin C, calyxin D displayed stable interactions and favorable binding energies. Experimental evaluation of drug targets and subsequent drug molecules designing against any target is time consuming and costly work. Therefore, the results of our study will greatly facilitate drug development process against RVFV. We acknowledge that computational analyses have certain limitations, thus further in-vitro and in-vivo studies are warranted to validate the inhibitory potential of selected promising candidates against RVFV.
